# Toxicity of Smokeless Tobacco Extract after 184-Day Repeated Oral Administration in Rats

**DOI:** 10.3390/ijerph13030281

**Published:** 2016-03-04

**Authors:** Chenlin Yu, Ziteng Zhang, Yangang Liu, Ying Zong, Yongchun Chen, Xiuming Du, Jikuai Chen, Shijie Feng, Jinlian Hu, Shufang Cui, Guocai Lu

**Affiliations:** 1 Department of Health Toxicology, College of Tropical Medicine and Public Health, Second Military Medical University, Shanghai 200433, China; ycl0408@163.com (C.Y.); cocoonzzt@163.com (Z.Z.); ll10421@163.com (Y.L.); standyup@hotmail.com (Y.Z.); chenyongchun1986@hotmail.com (Y.C.); duxiuming625888@sina.com (X.D.); cjk.smmu@hotmail.com (J.C.); fxiaojie1120@126.com (S.F.); robust_le@163.com (J.H.); 2 Laboratory Animal Centre, Second Military Medical University, Shanghai 200433, China

**Keywords:** smokeless tobacco extract, toxicology, nicotine

## Abstract

The use of smokeless tobacco (ST) is growing rapidly and globally. The consumption of ST is associated with an increased risk for developing chronic diseases, such as diabetes, hypercholesterolemia, and myocardial infarction, and has led to many public health problems. It is very important to access the toxicity of ST. This experiment presents data from 184-day toxicology studies in Sprague-Dawley (SD) rats designed to characterize the chronic effects of a smokeless tobacco extract (STE). The control group and treatment groups were matched for a range of nicotine levels. Animals were given STE by oral gavage with doses of 3.75 (low-dose), 7.50 (mid-dose) and 15.00 (high-dose) mg·nicotine/kg body weight/day for 184 days, followed by 30 days for recovery. Variables evaluated included body weights, feed consumption, clinical observations, clinical and anatomic pathology (including organ weights), and histopathology. Decreased body weights and organ weights (heart, liver and kidney) were found in animals in the mid-dose and high-dose groups. STE also showed moderate and reversible toxicity in esophagus, stomach, liver, kidney and lung.

## 1. Introduction

Worldwide, tobacco use is one of the most preventable causes of morbidity, disability and mortality [1,2,3]. Tobacco kills around six million people each year [4], accounting for 12% of global adult mortality [5]. Tobacco can be smoked or consumed in smokeless form. The use of smokeless tobacco (ST) is growing rapidly and globally [6]. Consumption of ST products is particularly popular in the United States, Sweden, India, Southeast Asia, South Asia, and various European countries [7], and now it is also gaining popularity in East Asia. According to the World Health Organization Framework Convention on Tobacco Control, weighted average prevalence rates of global ST use indicated that 23% of male adults and 7% of female adults currently use ST, and that 8% of boys and 6% of girls consume ST [8].

ST is a broad term that encompasses many different types of tobacco products used both orally and nasally. The two main forms of ST are snus and chewing tobacco [9]. Snus is finely ground tobacco usually placed between the gum and cheek, and chewing tobacco comes as loose leaf, plugs, or twists [10]. ST consumption causes cancers of the mouth, lip, nasal cavities, esophagus, and gut; diabetes; hypercholesterolemia; myocardial infarction; and adverse effects on pregnancy [11,12]. Thus, study of the toxicity of ST is very important.

The characterization and classification of ST products has been a continuously evolving process. However, the context and results vary between countries, research institutions, methodologies, research animal species and legal requirements [13]. Krautter *et al.* reported a 90-day toxicity study of tobacco ingestion in Sprague-Dawley rats in which all animals survived, and only slight changes in hematology and clinical chemistry were found, such as decreased body weights and feed consumption [14]. Theophilus *et al.* reported a toxicological evaluation of ST in Wistar Hannover rats for 90 days [15,16], confirming the reproducibility of the reductions in body/organ weights. However, little is known about the relative histopathological changes for potential toxicity in those studies. Avti *et al.* reported the effects of a short-term, high-dose and long-term, low-dose exposures to the smokeless tobacco extract (STE) on the antioxidant defense status and histopathological changes in liver, lung and kidney of male Wistar rats [17]. Willis *et al.* reported toxicity of an STE administered for three weeks on the reproductive system of adult male mice, reporting decreased levels of circulating testosterone, body weight and liver weight [10].

Our study was designed to further evaluate the toxicity of ST using an approach to simulate chronic daily exposure. Here, we evaluated three doses of ST exposure over a 184 day period and describe multiple toxic effects using a comprehensive analyses of histologic and clinical markers of toxicity. Given the prevalence of female children and adults that use smokeless tobacco, we conducted these analyses in male and female rats to help fill gaps in the current knowledge.

## 2. Materials and Methods

### 2.1. Experimental Animals and Housing Conditions

This study was conducted at the Center of Evaluation for Drug Safety, Second Military Medical University (SMMU, Shanghai, China). All protocols were approved by the Institutional Animal Care and Use Committee of SMMU (No. 20120025). Disease-free male (*n* = 80) and female (*n* = 80) SD rats were supplied by Sippr BK Laboratory Animal Ltd. (Shanghai, China). The body weight range at the start of treatment was 143.0–200.1 g for males and 138.0–166.2 g for females. They were housed for a 7-day acclimation period prior to the start of the experimental treatments. Animals were kept in a room maintained at 23 ± 2 °C, relative humidity of 40%–70%, and under a 12 h light/dark cycle.

### 2.2. Sample Preparation Procedures

An aqueous extract of smokeless tobacco (STE) (purity > 95.5%) was supplied by Shanghai Tobacco Group Co., Ltd. (Shanghai, China). STE powder was stored at 4 °C, protected from light and moisture. A nicotine standard (purity 99.0%, batch No. S18379725) was purchased from Toronto-Research-Chemicals, Inc. (TRC, Toronto, ON, Canada). An internal standard, fennel (purity 99.0%, batch No. ED4XO-ON) was purchased from J & K Chemical Ltd. (Shanghai, China). Mass spectroscopy grade acetonitrile, methanol, and formic acid were also purchased from J & K Chemical Ltd. (Shanghai, China). All other chemicals used were of the highest commercial grade available. The GC-MS analysis of nicotine was conducted at the College of Pharmacy, Second Military Medical University (Shanghai, China). For oral gavage, STE was suspended in distilled water at concentrations of 0.75, 1.50 and 3.00 mg·nicotine/mL and fresh samples were prepared once every three days.

### 2.3. Experimental Design

The experimental design used for the study is shown in Table 1. Male (*n* = 80) and female (*n* = 80) rats were divided into four groups at random by body weight. The control group (*n* = 40, C) was given dH_2_O by oral gavage. The STE low-dose group was given by gavage with a dose of 3.75 mg·nicotine/kg·BW/day (*n* = 40, L). The STE mid-dose group was given by gavage with a dose of 7.50 mg·nicotine/kg·BW/day (*n* = 40, M). The STE high-dose group was given by gavage with a dose of 15.00 mg·nicotine/kg·BW/day (*n* = 40, H). Each group included half male and half female rats.

Forty rats of each STE group (L, M and H) and the control group used in the 184-day toxicity test were given STE by gavage for 184 days (Weeks 1–26) and feed without STE to recover for 30 days (Weeks 27–31).Body weights of all rats were measured once weekly and the volume of STE were given by gavage to each rat was adjusted according to their body weights.

Rats were randomly selected and euthanized on Days 92, 184 and 214 (184 days of dosing and 30 days of recovery)of the study period. Blood was drawn via the abdominal aorta for clinical biochemistry, hematology, and coagulation testing. The absolute organs were weighed after blood collection. Histopathology examinations were performed after weighing.

### 2.4. Clinical Observations

Clinical signs were evaluated daily from the beginning of the acclimation period. Each animal was observed at least twice daily. Observations included skin, fur, and gait feature. Food consumption data and body weight data were collected once a week during the study period.

### 2.5. Clinical Test Parameters

#### 2.5.1. Hematology

Blood samples were collected in vacuum tube containers with ethylenediaminetetraacetic acid (EDTA). Hematological examination was performed using a Bayer ADVIA2120 automatic blood cell analyzer (Leverkusen, Germany) and a MERLIN MCLOplus (Leverkusen, Germany) to measure the parameters shown in Table 2.

#### 2.5.2. Serum Biochemistry

Blood for clinical chemistry was collected in vacuum tubes devoid of anticoagulant, allowed to clot at room temperature, centrifuged, and then serum was separated. The serum biochemical parameters shown in Table 2 were assessed using a HITACHI 7080 automated biochemical analyzer (Tokyo, Japan) and an Easylyte PLUS electrolyte analyzer (MEDICA, Bedford, MA, America).

### 2.6. Necropsy and Histopathology

A complete gross necropsy was conducted on all animals by visual inspection at the end of the exposure period (Day 184) and the recovery phase (Day 214). The following organs were trimmed, weighed and evaluated in terms of absolute weight and as a percentage of final body weight: brain, heart, kidney, liver, lung, spleen, thymus, testes, epididymis, uterus, ovaries, adrenals and thyroid. Paired organs were weighed together. The following tissues were preserved in 10% neutral buffered formalin: brain, pituitary gland, thyroid (including the parathyroid), trachea, heart, pancreas, spleen, adrenal glands, prostate, ovaries, uterus, esophagus, duodenum, jejunum, ileum, cecum, colon, rectum, mesenteric lymph nodes, sub maxillary lymph nodes, aorta, eyes, skeletal muscle, sciatic nerve, mammary gland, sternum, salivary glands, spinal cord, urinary bladder, lung (including the bronchi), liver, kidneys, stomach, bone marrow (sternal), thymus, sternum, and any gross lesions or masses. The lung tissue was inflated with fixative at the time of necropsy. Testes and epididymides were preserved in Davidson’s fixing solution for 24 h, and then in 10% neutral buffered formalin. All the preserved tissues were paraffin embedded, sectioned, stained with hematoxylin and eosin and examined microscopically. Bone marrow cellular morphology examination was conducted using both paraffin-embedded sternum sections (sternal) and smears.

### 2.7. Statistical Analysis

All measurements are expressed as the mean ± standard deviation. For each sex, food consumption, body weight, organ weight, hematological parameters and clinical chemistry data were analyzed by parametric one-way analysis using the F-test (ANOVA, two-sided) with Statistical Product and Service Solutions (SPSS) v11.5 (IBM: Armonk, America). If the resulting *p*-value was <0.05, a comparison of each group using the LSD test was performed for the hypothesis of equal means. When the data failed to follow a normal distribution test even after being converted, non-parametric one-way analysis using the Kruskal–Wallis test was done. The Dunnett T3 test was applied when the data could not be assumed to follow homogeneity variance.

## 3. Results

### 3.1. Clinical Observations

**Figure 1:**
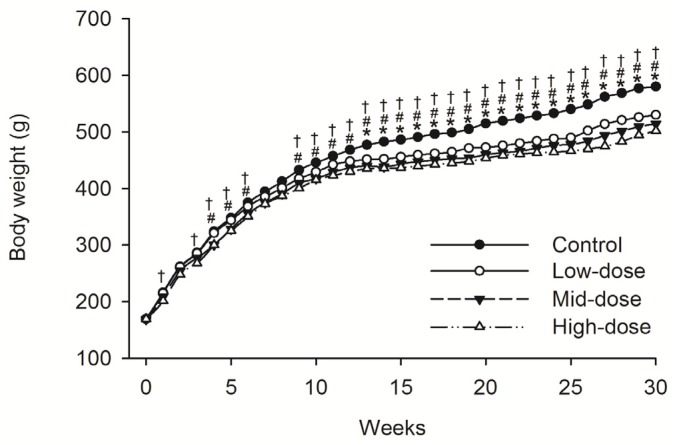
Mean body weight trend chart of male SD rats treated with STE. * *p* < 0.05 *vs.* Low-dose group. ^#^ *p* < 0.05 *vs.* Mid-dosegroup. ^†^ *p* < 0.05 *vs.* High-dosegroup. Note: C = control group (distilled water), L = low-dose group (STE 3.75 mg/kg), M = mid-dose group (STE 7.50 mg/kg), H = high-dose group (STE 15.00 mg/kg).

**Figure 2:**
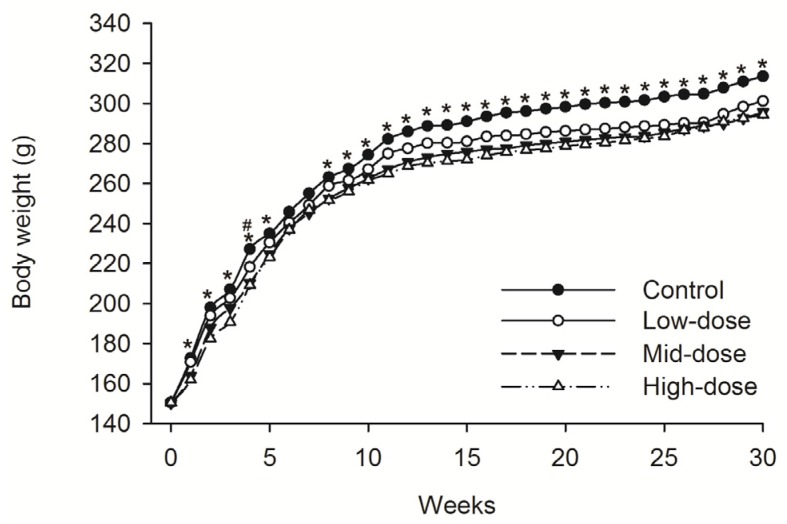
Mean body weight trend chart of female SD rats treated with STE. ^#^ *p* < 0.05 *vs.* Low-dose group. * *p* < 0.05 *vs.* Mid-dose group and High-dose group. Note: C = control group (distilled water), L = low-dose group (STE 3.75 mg/kg), M = mid-dose group (STE 7.50 mg/kg), H = high-dose group (STE 15.00 mg/kg).

No animals died during dosing phase. Mean food consumption data indicated no treatment-related changes during the dosing phase or the recovery phase. A difference in the body weight was observed intermittently in male animals given low-dose STE (Weeks 13–30, *p* < 0.05), mid-dose STE (Weeks 4–6 and 9–30, *p* < 0.05) and high dose STE (Weeks 1, 3–6 and 9–30, *p* < 0.05), compared with controls (Figure 1). Body weights were also significantly decreased in females given the low (Weeks 4, *p* < 0.05), medium (Weeks 1–5 and 8–30, *p* < 0.05) and high (Weeks 1–5 and 8–30, *p* < 0.05) doses of STE, compared with controls (Figure 2).

### 3.2. Clinical Test Parameters

#### 3.2.1. Hematology

Hematology results are shown in Table 3 and Table 4. Slight decrease were observed in the number of eosinophils (EOS) in the male STE-treated rats (all doses) compared with control-treated rats on Day 92 of dosing, however, the EOS counts were within the normal range. Similar results were observed for EOS in female rats given high-dose STE. The WBC was increased in female STE-treated rats in the high-dose groups compared to the control-treated rats on Day 92. These values were also within the normal range.

#### 3.2.2. Serum Biochemistry

Serum chemistry parameters are shown in Table 5 and Table 6. Levels of ALT and TBIL were significantly increased in male rats from the high-dose group compared to controls on Day 184 (*p* < 0.05). The mean ALT was 2.36 times higher than the controls and the mean TBIL was 1.59 times higher than that of the controls. Significantly increased TP concentrations were observed in male rats in all dose-groups, compared to the control group on Day 184 (*p* < 0.05). ALB levels were significantly increased in male rats of all groups compared to the controls (*p* < 0.05) on Day 184. Significantly increased BU levels were observed in male rats of all groups compared to the control group on Days 92, 184 and 214 (*p* < 0.05).

In female rats, ALT levels were significantly increased in the high-dose group compared with the control group on Day 92 (*p* < 0.05), and in the mid-dose and high-dose group on Day 184 (*p* < 0.05). Increased levels of TBIL were observed in the high-dose group, which were 1.47 times of the control group, and a significant increase in CREA was observed in the high-dose group on Day 92 (*p* < 0.05). TCH was increased at all doses STE on Day 184 (*p* < 0.05), and BU levels were increased level in the high-dose group compared to the control group on Day 92 and Day 184.

### 3.3. Necropsy and Histopathlolgoy

#### 3.3.1. Organ Weights

Absolute organ weights are given in Table 7 and Table 8. A significant decrease in absolute organ weight of heart was observed in both male and female rats of the high-dose group compared with the control group on Days 92 and 184 (*p* < 0.05). A significant decrease in absolute organ weight of liver was observed in the male rats of the high-dose group, and in the female rats of the mid-dose and high-dose group compared with the control group on Days 92 and 184 (*p* < 0.05). A significant decrease in absolute organ weight of kidney was observed in both male and female rats of the high-dose group compared with the control group on Days 92 and 184 (*p* < 0.05). A significant decrease in absolute organ weight of brain and thymus was observed in the male rats of the high-dose group compared with the control group on Day 184 (*p* < 0.05).

#### 3.3.2. Histopathologic Findings

**Figure 3:**
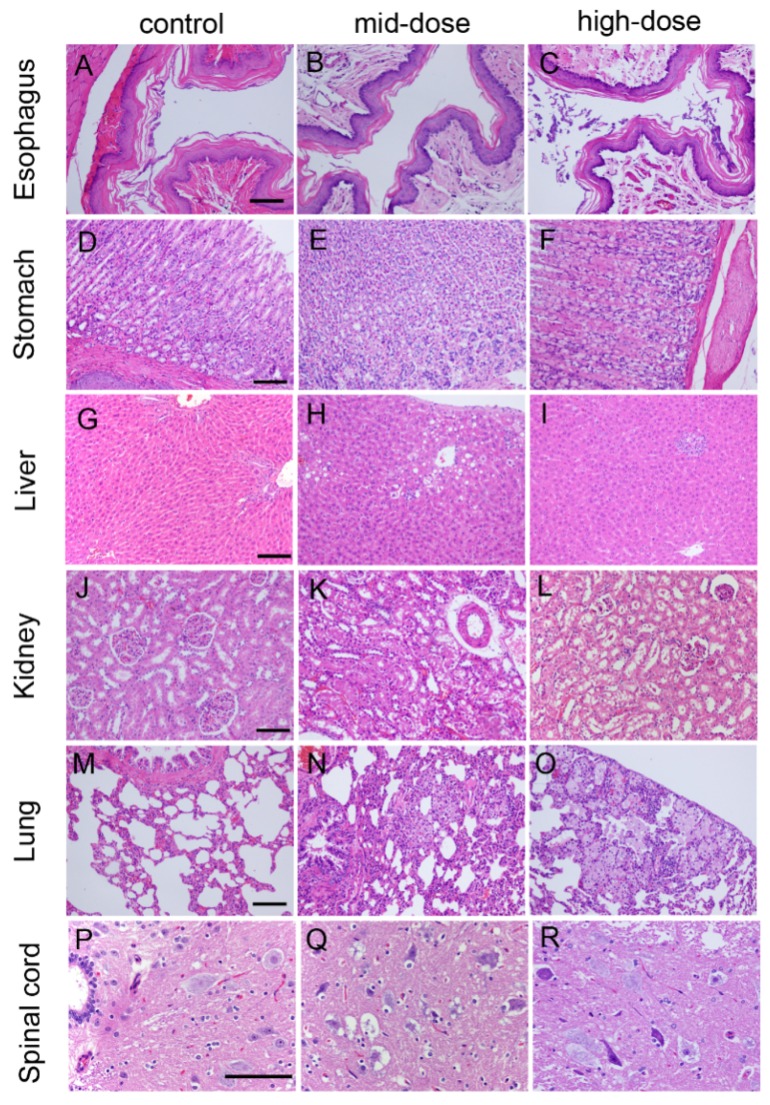
Hematoxylin and eosin-stained histologic sections of rat esophagus (**A**–**C**); stomach (**D**–**F**); liver (**G**–**I**); kidney (**J**–**L**); lung (**M**–**O**); and spinal cord (**P**–**R**). Note: (**A**) Esophagus from control rat; (**B**) esophagus from mid-dose rat; (**C**) esophagus from high-dose rat; (**D**) stomach from control rat; (**E**) stomach from mid-dose rat; (**F**) stomach from high-dose rat; (**G**) liver from control rat; (**H**) liver from mid-dose rat; (**I**) liver from high-dose rat; (**J**) kidney from control rat; (**K**) kidney from mid-dose rat; (**L**) kidney from high-dose rat; (**M**) lung from control rat; (**N**) lung from mid-dose rat; (**O**) lung from high-dose rat; (**P**) spinal cord from control rat; (**Q**) spinal cord from mid-dose rat; and(**R**) spinal cord from high-dose rat. Scale bar: 25 µm.

Histopathologic findings of rats in all dose groups are shown in Table 9 and Figure 3.

Sixteen cases of keratinized stratified squamous epithelium or basophilic material attached with stratified squamous epithelium were observed in all dose groups on Day 92, and there were twenty-three cases having these conditions in all dose groups on Day 184.

Three cases of slight or moderate gastric epithelial degeneration were observed in the high-dose group on Day 92, 12 cases of slight or moderate gastric epithelial degeneration were observed in high-dose group on Day 184, and two cases of slight gastric epithelial degeneration were observed in high-dose group on Day 214.

Twenty-three cases of slight or moderate chronic inflammatory cell infiltrates, hepatocellular degeneration or necrosis were observed in all STE groups on Day 92. Thirty-six cases of slight or moderate chronic inflammatory cell infiltrates, hepatocellular degeneration or necrosis were observed in all STE groups on Day 184, and nine cases of slight chronic inflammatory cell infiltrates, hepatocellular degeneration or necrosis were observed in all dose-group on Day 214.

Eight cases of foam cell focal in lung were observed in all dose-group on Day 92, and 19 cases of foam cell foci in the lung were observed in all treatment groups on Day 184.

Twenty cases of kidney renal proximal tubule epithelium (slight to mild degeneration) were observed in all dose groups on Day 184. Two females in the high-dose group also had proximal tubule degeneration accompanied by mild glomerular vascular loop collapse, and glomerular atrophic changes. Only one case of focal inflammatory cells infiltration was observed on Day 214.

Thirty-one cases of gray matter neuronal cell body degeneration in the spinal cord were observed in STE-treated rats, showing swelling of the nerve cell body, fluid accumulation within cells, plasma cells with small vacuoles, small or dissolved perinuclear nissl, gray cytoplasm, severe nerve cell degeneration, Nissl body disappearance, and membrane rupture.

## 4. Discussion

Overall, the toxicity we observed following STE administration was observed at the high and medium doses. No animals died during dosing or the recovery period. A dose-dependent reduction in body weight was observed in all treated animals, with statistically significant toxicity reductions in the medium and high dose groups of male and female rats. Other indications of obvious tissue pathological changes were observed in the digestive system (mainly the liver and stomach), the urinary system (kidney) and the respiratory system (lungs). Such effects suggest that use of ST produces organ system toxicity.

As the use of ST is to chew or to hold in the mouth, it is known that ST can induce changes in the oral mucosa associated with oral injury, inflammation and leukoplakia [18,19,20,21]. ST exposure can also induce gingival recession with associated attachment loss, cervical abrasion, and damage to the oral tissues [22]. In the present study, animals were orally administered STE by oral gavage. In the control group, four cases of keratinized stratified squamous epithelium were found on the esophageal mucosa, which maybe the mechanical damage of oral gavage, and the subcutaneous connective tissue showed no abnormalities. In all dose groups, basophilic secretions were observed on the surface of the stratified squamous epithelium of the esophageal mucosa and epithelial keratoses were irregular with unequal thickness. These results indicate that ST had a slight effect on mucous membrane of the esophagus, consistent with results from previous studies [18,19,20,21,22].

We also observed slight or moderate gastric epithelial degeneration in high-dose group. The glandular epithelium of stomach lining had mild swelling, cytoplasmic cavitation, karyopyknosis or degeneration in the mid-dose or high-dose group. These changes were less pronounced during the recovery days, which indicated that the gavage of STE might have had a localized effect in the gastrointestinal tract. It has been shown both in epidemiological and experimental studies that smoking has harmful effects on the gastric mucosa [23,24,25,26], and is also a risk factor for peptic ulcer disease [27]. Users of smokeless or chewing tobacco have been shown to have higher death rates from cancer of the gastrointestinal tract compared with non-users [28].

Liver enzymes are normally found within the cells of the liver. It is well known that when the liver is injured or damaged, the liver enzymes such as ALT, AST and ALP are released into the blood [29]. Elevated bilirubin levels can be indicative of liver disorders or blockage of bile ducts. Increased serum AST, ALT and TBIL level are important markers of liver injury, attributing to the damaged structural integrity of the liver [30]. ALT is primarily found in the liver, making it a more specific test for detecting liver abnormalities [31]. Increased serum ALT and TBIL levels with related abnormalities in liver histology were found during the dosing phase, which decreased in the high-dose group during the recovery phase. Likewise, focal inflammatory infiltrates in the liver in the high-dose group, and cell and hepatic steatosis in the mid-dose group began to ameliorate during the recovery period. These findings indicated that STE had a moderate and reversible effect on liver function.

Arimilli *et al.* demonstrated that extract of ST caused DNA damage and IL-8 production in cultured human cells [32]. Dygert [33] demonstrated that multiple bacterial strains found in ST raises the possibility of chronic exposure to infectious agents as a mechanism for the development of chronic lung inflammation. Kumar *et al.* demonstrated that administration of aqueous extract of ST impairs the enzymatic antioxidant defense system, reduces glutathione levels in lung, liver and kidney, and caused moderate infiltration of phagocytic cells in the liver and lung [17]. Similarly, we observed foam cell foci in the alveolar lumen in all treated groups, which are a characteristic of interstitial inflammation [34]. These findings indicate that STE had a mild toxic effect on the lungs.

It is well known that one of the primary functions of the kidneys is to remove creatinine, which is the waste product of muscle breakdown, from the bloodstream. High levels of creatinine can indicate kidney failure, which can be temporary or permanent [35]. Creatinine is commonly measured as an index of glomerular function [36]. Urea is a byproduct from protein breakdown. About 90% of urea produced is excreted through the kidney [37], and the blood urea nitrogen (BU) test is also used to determine if the kidneys are successfully filtering the blood. Urea nitrogen is normal in the blood at small levels, but higher levels may indicate that the individual is experiencing kidney problems [35].

We observed evidence of kidney toxicity following STE administration, with increased serum BU and Crea levels and related histopathology changes during the dosing phase. The levels of BU and Crea remained significantly elevated in the high-dose group during the recovery phase. Kumar *et al.* noted that administration of an aqueous extract of ST significantly decreased the level of hepatic glutathione (GSH), glutathione peroxidase (GPx), superoxide dismutase (SOD) and catalase (CAT) of the liver, lung and kidney, with a slight to moderate degree of histopathological abnormalities in the liver and lung; however, similar histopathological changes in the kidney were not found [17]. In the present study, proximal tubule epithelium of renal cortex had mild degeneration and swelling. The glomerular vascular loops showed a slight atrophy, with reduced cell numbers and smaller cells. In the renal medulla, moderate histopathology changes were observed, with collecting duct occlusion and hardening. These pathological changes in the kidney were not as severe during the recovery phase, indicating that the effect on kidney function was reversible to an extent.

## 5. Conclusions

The toxic effects of STE we observed were decreased body weights in animals from the mid-dose and high-dose groups. Meanwhile, STE had a moderate and reversible toxic effect on the esophagus, stomach, liver, kidney and lung. These findings have identified important dose-related toxic effects that provide the basis for further mechanistic studies. As use of ST has become a worldwide concern for human health because of its increasing adverse effects [17], an understanding of the toxicity of STE will have important public health implications.

## Figures and Tables

**Table 1:** 184-Day rat study designs.

Number	Group	Dose Group Abbreviations	Target Dosage of Nicotine (mg/kg/Day)	Number Animals/Group					
				92 Days		184 Days		214 Days	
				M	F	M	F	M	F
1	Control	C	0	5	5	10	10	5	5
2	Aqueous extract of smokeless tobacco in low dose	L	3.75	5	5	10	10	5	5
3	Aqueous extract of smokeless tobacco in medium dose	M	7.50	5	5	10	10	5	5
4	Aqueous extract of smokeless tobacco in high dose	H	15.00	5	5	10	10	5	5

Notes: M = Males; F = Females.

**Table 2:** Parameters evaluated in hematology and serum chemistry.

Hematology	Serum Chemistry
Erythrocyte count (RBC)	Alanine aminotransferase (ALT)
Hemoglobin concentration (HB)	Aspartate aminotransferase (AST)
Hematocrit (HCT)	Alkaline phosphatase (ALP)
Mean corpuscular volume (MCV)	Glucose (GLU)
Mean corpuscular hemoglobin (MCH)	Total protein (TP)
Mean corpuscular hemoglobin concentration (MCHC)	Albumin (ALB)
Platelet (PLT)	Urea nitrogen (BU)
Total leukocyte count (WBC)	Creatinine (CREA)
Lymphocytes (LYMPH)	Triglyceride (TG)
Monocytes (MONO)	Total cholesterol (TCH)
Eosinophils (EOS)	Total bilirubin (TBIL)
Basophils (BASO)	Gamma-glutamyltransferase (GGT)
Neutrophils (NEUT)	Calcium (CA)
Reticulocyte count (RETIC)	Phosphorus (P)
Prothrombin time (PT)	Creatine phosphokinase (CK)
Activated partial thromboplastin time (APTT)	Lactate dehydrogenase (LDH)
Thromboplastin time (TT)	Amylase (AMY)
Fibrinogen (FIB)	Lipase (LIP)
	K^+^, Na^+^, Cl^−^

**Table 3:** Summary of hematology in male rats ($\overset{–}{x}$ ± SD, d92 *n* = 5, d184 *n* = 10, d214 *n* = 5).

Items	Days	C	L	M	H
RBC (×10^12^ L^−1^)	d92	8.47 ± 0.34	8.25 ± 0.44	8.19 ± 0.21	8.30 ± 0.35
	d184	8.63 ± 0.55	8.30 ± 0.44	8.39 ± 0.38	8.16 ± 0.54
	d214	8.00 ± 0.97	8.80 ± 0.41	8.91 ± 0.31	8.33 ± 0.55
HGB (g·L^−1^)	d92	147 ± 4	154 ± 6	144 ± 4	144 ± 5
	d184	142 ± 5	139 ± 5	143 ± 4	140 ± 6
	d214	142 ± 8	142 ± 5	144 ± 3	143 ± 9
HCT (%)	d92	45.5 ± 3.4	49.1 ± 1.5	46.1 ± 1.4	44.2 ± 3.6
	d184	43.8 ± 1.7	43.1 ± 1.5	44.1 ± 1.2	42.4 ± 2.1
	d214	41.4 ± 4.8	45.5 ± 0.6	45.5 ± 2.5	44.1 ± 4.4
MCV (fl)	d92	53.8 ± 4.4	54.4 ± 1.7	56.2 ± 0.7	53.4 ± 5.1
	d184	50.9 ± 1.7	52.0 ± 1.7	52.6 ± 1.9	52.1 ± 2.0
	d214	51.8 ± 0.8	51.7 ± 1.8	51.0 ± 1.1	52.9 ± 2.5
MCH (pg)	d92	17.4 ± 0.4	17.0 ± 0.5	17.6 ± 0.2	17.3 ± 0.4
	d184	16.5 ± 0.6	16.8 ± 0.7	17.1 ± 0.6	16.8 ± 0.7
	d214	17.9 ± 2.7	16.1 ± 0.8	16.1 ± 0.5	17.2 ± 0.6
MCHC (g·L^−1^)	d92	325 ± 26	314 ± 5	313 ± 4	327 ± 31
	d184	324 ± 4	324 ± 4	324 ± 4	322 ± 4
	d214	346 ± 49	312 ± 14	316 ± 17	326 ± 24
PLT (×10^9^ L^−1^)	d92	1041 ± 115	988 ± 107	1023 ± 57	1150 ± 166
	d184	1097 ± 63	1056 ± 60	1041 ± 107	1057 ± 161
	d214	1066 ± 163	1000 ± 98	1050 ± 91	997 ± 74
WBC (×10^9^ L^−1^)	d92	4.83 ± 2.09	5.63 ± 1.51	4.69 ± 0.88	5.25 ± 0.69
	d184	5.19 ± 1.02	5.13 ± 1.34	4.72 ± 1.25	4.65 ± 1.06
	d214	4.24 ± 0.43	3.88 ± 1.11	4.00 ± 0.44	4.81 ± 1.73
NEUT (%)	d92	22.3 ± 4.5	33.1 ± 12	27.1 ± 6.7	21.8 ± 4.9
	d184	27.5 ± 8.1	37.6 ± 8.5 *	31.8 ± 10.7	27.7 ± 5.8
	d214	27.2 ± 6.3	28.0 ± 10.8	30.5 ± 7.2	27.4 ± 9.5
LYMPH (%)	d92	73.1 ± 5.0	61.8 ± 10.9	68.9 ± 6.9	74.1 ± 5.0
	d184	68.6 ± 7.8	58.7 ± 8.6 *	64.9 ± 10.5	68.8 ± 6.2
	d214	67.6 ± 6.2	66.8 ± 11.2	64.4 ± 7.3	68.2 ± 10.3
MONO (%)	d92	1.9 ± 0.2	3.1 ± 1.3	2.0 ± 0.7	2.4 ± 0.8
	d184	2.0 ± 0.8	2.2 ± 0.7	2.0 ± 0.7	2.0 ± 0.7
	d214	2.6 ± 0.5	2.6 ± 0.4	2.9 ± 0.7	2.4 ± 0.6
EOS (%)	d92	2.3 ± 0.6	2.0 ± 0.2 *	1.8 ± 0.4 *	1.6 ± 0.3 *
	d184	1.7 ± 0.7	1.6 ± 0.7	1.5 ± 0.4 *	1.4 ± 0.8 *
	d214	2.4 ± 0.5	2.3 ± 0.6	1.9 ± 0.4 *	1.7 ± 0.6 *
BASO (%)	d92	0.1 ± 0.1	0.2 ± 0.1	0.1 ± 0.1	0.2 ± 0.1
	d184	0.1 ± 0.1	0.1 ± 0.1	0.1 ± 0.1	0.1 ± 0.1
	d214	0.1 ± 0.1	0.1 ± 0.0	0.1 ± 0.0	0.0 ± 0.1
RETIC (%)	d92	2.36 ± 0.50	2.26 ± 0.44	1.80 ± 0.19	2.44 ± 0.25
	d184	1.97 ± 0.34	2.14 ± 0.43	1.97 ± 0.23	1.72 ± 0.48
	d214	1.53 ± 0.16	1.43 ± 0.31	1.43 ± 0.31	1.71 ± 0.17
PT (s)	d92	14.7 ± 0.8	14.3 ± 0.3	14.6 ± 0.6	14.7 ± 0.3
	d184	13.6 ± 3.2	13.4 ± 2.9	13.8 ± 1.9	14.6 ± 0.7
	d214	15.7 ± 1.2	15.6 ± 1.1	14.7 ± 0.9	15.2 ± 0.5
APTT (s)	d92	19.0 ± 1.3	18.9 ± 1.6	19.2 ± 2.1	18.2 ± 1.7
	d184	17.7 ± 2.7	16.3 ± 2.4	16.1 ± 3.0	14.5 ± 1.8
	d214	15.8 ± 1.5	18.7 ± 2.7	14.2 ± 3.5	13.9 ± 2.2
TT (s)	d92	22.3 ± 1.9	21.9 ± 1.6	21.5 ± 2.4	20.2 ± 0.8
	d184	19.3 ± 0.7	19.2 ± 0.7	19.2 ± 0.5	18.8 ± 0.6
	d214	21.6 ± 0.7	21.7 ± 1.7	21.9 ± 1.0	21.5 ± 1.3
FIB (mg/dL)	d92	2.20 ± 0.20	2.30 ± 0.30	2.10 ± 0.10	2.00 ± 0.10
	d184	2.25 ± 0.26	2.28 ± 0.13	2.29 ± 0.40	2.44 ± 1.19
	d214	2.18 ± 0.22	2.27 ± 0.20	2.15 ± 0.12	2.04 ± 0.15

Notes: * A significant difference at *p* < 0.05 level compared with the control group. C = control group (distilled water), L = low-dose group (STE 3.75 mg/kg), M = mid-dose group (STE 7.50 mg/kg), H = high-dose group (STE 15.00 mg/kg).

**Table 4:** Summary of hematology in female rats ($\overset{–}{x}$ ± SD, d92 *n* = 5, d184 *n* = 10, d214 *n* = 5).

Items	Days	C	L	M	H
RBC (×10^12^ L^−1^)	d92	8.03 ± 0.53	7.24 ± 0.54	7.32 ± 0.40	7.49 ± 0.52
	d184	7.50 ± 0.49	7.20 ± 0.26	7.56 ± 0.48	7.60 ± 0.56
	d214	7.73 ± 0.20	7.34 ± 0.34	7.30 ± 1.05	6.98 ± 0.89
HGB (g·L^−1^)	d92	145 ± 8	138 ± 5	136 ± 6	138 ± 5
	d184	134 ± 6	130 ± 6	131 ± 6	131 ± 7
	d214	129 ± 9	129 ± 5	127 ± 7	131 ± 5
HCT (%)	d92	46.1 ± 2.7	42.0 ± 3.2	41.5 ± 3.8	43.5 ± 1.0
	d184	41.1 ± 1.9	39.9 ± 1.9	40.3 ± 2.0	40.7 ± 2.1
	d214	42.7 ± 1.2	40.8 ± 1.8	39.6 ± 5.3	38.2 ± 4.5
MCV (fl)	d92	57.6 ± 2.1	58.1 ± 2.4	56.8 ± 5.5	58.4 ± 4.1
	d184	54.9 ± 2.1	55.5 ± 1.4	53.4 ± 1.6	53.7 ± 1.6
	d214	55.2 ± 0.8	55.6 ± 1.9	54.3 ± 1.2	54.7 ± 1.2
MCH (pg)	d92	18.0 ± 0.5	19.0 ± 0.9	18.6 ± 0.5	18.5 ± 0.8
	d184	17.8 ± 0.6	18.0 ± 0.4	17.3 ± 0.5	17.3 ± 0.5
	d214	16.6 ± 1.1	17.6 ± 1.2	17.8 ± 3.7	19.1 ± 2.7
MCHC (g·L^−1^)	d92	314 ± 5	328 ± 21	330 ± 37	317 ± 13
	d184	325 ± 5	324 ± 3	325 ± 3	323 ± 4
	d214	302 ± 19	317 ± 24	326 ± 63	348 ± 49
PLT (×10^9^ L^−1^)	d92	1127 ± 95	1225 ± 218	1048 ± 278	1019 ± 95
	d184	1104 ± 133	1056 ± 102	1098 ± 90	1106 ± 87
	d214	1153 ± 173	1106 ± 170	1128 ± 145	1102 ± 106
WBC (×10^9^ L^−1^)	d92	3.21 ± 0.76	3.83 ± 0.85	4.33 ± 0.97	5.30 ± 0.98 *
	d184	2.54 ± 0.66	3.03 ± 0.89	3.58 ± 2.13	4.81 ± 2.48
	d214	3.65 ± 1.43	2.94 ± 1.10	2.53 ± 0.74	2.39 ± 0.53
NEUT (%)	d92	14.9 ± 5.8	11.8 ± 2.9	16.2 ± 3.4	15.2 ± 6.3
	d184	17.7 ± 7.3	20.2 ± 14.0	15.0 ± 4.5	18.2 ± 7.9
	d214	22.0 ± 7.7	17.1 ± 3.2	19.5 ± 7.6	15.3 ± 5.7
LYMPH (%)	d92	81.1 ± 5.6	83.8 ± 3.3	80.2 ± 2.8	81.6 ± 6.5
	d184	78.3 ± 8.7	76.0 ± 14.5	81.3 ± 4.9	78.3 ± 7.7
	d214	73.4 ± 8.3	77.8 ± 3.8	75.0 ± 6.8	80.9 ± 6.7
MONO (%)	d92	1.4 ± 0.3	2.1 ± 0.8	1.9 ± 0.8	1.7 ± 0.2
	d184	1.8 ± 1.3	2.0 ± 0.9	1.7 ± 0.4	2.1 ± 0.8
	d214	2.3 ± 0.4	2.5 ± 1.0	2.5 ± 0.9	1.5 ± 0.6
EOS (%)	d92	2.1 ± 0.7	1.8 ± 0.5 *	1.6 ± 0.3 *	1.5 ± 0.2 *
	d184	1.8 ± 0.6	1.5 ± 0.6 *	1.4 ± 0.6 *	1.3 ± 0.4 *
	d214	2.0 ± 0.6	2.3 ± 0.5	2.4 ± 0.7	2.0 ± 0.5
BASO (%)	d92	0.3 ± 0.1	0.2 ± 0.1	0.2 ± 0.1	0.2 ± 0.1
	d184	0.1 ± 0.1	0.0 ± 0.1	0.1 ± 0.1	0.1 ± 0.1
	d214	0.1 ± 0.0	0.1 ± 0.1	0.1 ± 0.1	0.1 ± 0.0
RETIC (%)	d92	2.28 ± 0.47	2.12 ± 0.22	2.50 ± 0.16	2.80 ± 0.79
	d184	1.72 ± 0.43	1.89 ± 0.18	1.62 ± 0.36	1.60 ± 0.35
	d214	1.53 ± 0.38	1.40 ± 0.21	1.75 ± 0.24	1.53 ± 0.25
PT (s)	d92	13.9 ± 0.5	13.8 ± 0.3	14.1 ± 0.1	14.4 ± 0.5
	d184	14.0 ± 0.4	13.8 ± 0.4	13.9 ± 0.7	14.6 ± 1.0
	d214	14.3 ± 0.7	14.6 ± 0.8	14.6 ± 0.9	14.9 ± 0.8
APTT (s)	d92	17.8 ± 0.6	17.5 ± 0.7	17.5 ± 0.7	17.4 ± 1.0
	d184	15.8 ± 2.6	15.5 ± 2.0	15.6 ± 1.6 *	14.5 ± 2.4 *
	d214	12.4 ± 1.4	12.9 ± 1.6	13.0 ± 1.7	11.8 ± 1.1
TT (s)	d92	21.8 ± 1.2	22.0 ± 1.5	21.3 ± 1.3	20.5 ± 1.8
	d184	19.1 ± 0.6	19.1 ± 1.0	19.2 ± 0.9	18.9 ± 0.5
	d214	21.5 ± 1.4	22.5 ± 2.6	21.5 ± 0.8	21.8 ± 1.1
FIB (mg/dL)	d92	1.71 ± 0.08	1.80 ± 0.27	1.89 ± 0.16	1.86 ± 0.21
	d184	1.67 ± 0.12	1.69 ± 0.12	1.83 ± 0.43	1.72 ± 0.20
	d214	1.74 ± 0.22	1.55 ± 0.19	1.71 ± 0.26	1.67 ± 0.09

Notes: * A significant difference at *p* < 0.05 level compared with the control group. C = control group (distilled water), L = low-dose group (STE 3.75 mg/kg), M = mid-dose group (STE 7.50 mg/kg), H = high-dose group (STE 15.00 mg/kg).

**Table 5:** Summary of serum biochemistry in male rats ($\overset{–}{x}$ ± SD, d92 *n* = 5, d184 *n* = 10, d214 *n* = 5).

Items	Days	C	L	M	H
ALT (nmol·s^−1^·L^−1^)	d92	912 ± 111	1030 ± 290	941 ± 96	1004 ± 92
	d184	866 ± 134	841 ± 95	931 ± 105	2040 ± 1832 *
	d214	794 ± 65	759 ± 36	900 ± 261	978 ± 162
AST (nmol·s^−1^·L^−1^)	d92	2325 ± 535	2235 ± 197	2356 ± 407	2072 ± 273
	d184	2559 ± 458	2226 ± 223	2226 ± 213	3101 ± 1692
	d214	1907 ± 238	2107 ± 567	2176 ± 566	2392 ± 224
TP (g·L^−1^)	d92	59.6 ± 2.6	59.2 ± 2.6	57.1 ± 2.0	56.9 ± 1.3
	d184	60.7 ± 1.6	57.1 ± 1.7 *	58.2 ± 2.1 *	56.3 ± 3.2 *
	d214	58.5 ± 1.7	57.5 ± 2.1	57.3 ± 1.8	57.4 ± 2.8
ALB (g·L^−1^)	d92	34.8 ± 1.3	34.3 ± 1.0	34.4 ± 1.0	34.7 ± 0.9
	d184	37.0 ± 0.6	35.2 ± 0.9 *	36.4 ± 1.1	35.8 ± 1.5 *
	d214	36.0 ± 0.9	35.7 ± 1.5	35.9 ± 0.6	36.0 ± 1.4
TBIL (μmol·L^−1^)	d92	1.76 ± 0.50	2.05 ± 0.83	2.42 ± 1.06	2.95 ± 1.24
	d184	1.99 ± 0.74	2.72 ± 0.89	2.89 ± 1.20	3.17 ± 1.87 *
	d214	2.06 ± 0.46	2.16 ± 0.96	2.06 ± 1.05	2.54 ± 1.06
GLU (mmol·L^−1^)	d92	7.26 ± 0.86	6.88 ± 1.09	6.70 ± 1.11	6.94 ± 0.39
	d184	7.32 ± 0.68	6.75 ± 0.64	6.99 ± 0.57	6.87 ± 0.84
	d214	7.70 ± 0.46	7.33 ± 1.03	7.08 ± 0.99	6.85 ± 0.43
BU (mmol·L^−1^)	d92	5.89 ± 0.50	6.73 ± 0.82 *	6.61 ± 0.63	7.75 ± 0.25 *
	d184	6.16 ± 0.68	6.48 ± 0.79	6.76 ± 1.03	8.97 ± 1.26 *
	d214	6.01 ± 0.81	6.69 ± 0.89	6.55 ± 0.76	8.24 ± 1.15 *
CREA (μmol·L^−1^)	d92	30.6 ± 2.8	34.0 ± 5.4	29.6 ± 4.8	31.8 ± 4.8
	d184	33.3 ± 3.0	32.3 ± 4.0	30.1 ± 3.7	33.0 ± 3.5
	d214	28.0 ± 3.1	34.2 ± 6.2	29.2 ± 6.4	34.1 ± 4.3
Ca (mmol·L^−1^)	d92	2.2 ± 0.1	2.3 ± 0.1	2.2 ± 0.0	2.3 ± 0.1
	d184	2.4 ± 0.1	2.3 ± 0.0	2.4 ± 0.1	2.4 ± 0.1
	d214	2.7 ± 0.1	2.6 ± 0.1	2.6 ± 0.0	2.6 ± 0.1
P (mmol·L^−1^)	d92	1.90 ± 0.18	1.87 ± 0.17	2.02 ± 0.14	2.01 ± 0.10
	d184	1.77 ± 0.18	1.80 ± 0.10	1.90 ± 0.12	2.07 ± 0.31 *
	d214	1.79 ± 0.14	1.71 ± 0.07	1.81 ± 0.10	1.82 ± 0.18
TCH (mmo·L^−1^)	d92	1.36 ± 0.27	1.43 ± 0.42	1.17 ± 0.16	1.23 ± 0.37
	d184	1.54 ± 0.37	1.61 ± 0.51	1.45 ± 0.26	1.31 ± 0.36
	d214	1.47 ± 0.13	1.37 ± 0.29	1.30 ± 0.13	1.21 ± 0.26
TG (mmol·L^−1^)	d92	0.43 ± 0.16	0.54 ± 0.33	0.31 ± 0.08	0.27 ± 0.07
	d184	0.61 ± 0.16	0.51 ± 0.10	0.51 ± 0.07	0.47 ± 0.20
	d214	0.61 ± 0.16	0.59 ± 0.15	0.66 ± 0.13	0.52 ± 0.11
CK (μmol·s^−1^·L^−1^)	d92	7.03 ± 2.27	6.15 ± 2.43	6.7 ± 2.45	5.45 ± 1.70
	d184	10.09 ± 2.81	8.73 ± 1.94	8.67 ± 2.10	8.59 ± 2.24
	d214	7.17 ± 2.34	8.76 ± 3.98	8.57 ± 3.87	9.82 ± 2.75
LDH (μmol·s^−1^·L^−1^)	d92	24.33 ± 9.21	20.74 ± 8.24	23.91 ± 8.56	20.92 ± 5.99
	d184	28.69 ± 7.39	24.69 ± 4.93	23.63 ± 5.14	23.23 ± 6.91
	d214	17.83 ± 6.86	22.15 ± 9.67	21.25 ± 8.60	23.13 ± 4.54
ALP (μmol·s^−1^·L^−1^)	d92	1.36 ± 0.30	1.48 ± 0.32	1.81 ± 0.18	2.13 ± 0.62
	d184	1.27 ± 0.21	1.31 ± 0.40	1.49 ± 0.31	2.03 ± 0.52
	d214	1.27 ± 0.31	1.31 ± 0.09	1.53 ± 0.30	1.31 ± 0.20
K (mmol·L^−1^)	d92	4.62 ± 0.31	4.69 ± 0.19	4.86 ± 0.46	4.72 ± 0.18
	d184	4.59 ± 0.16	4.52 ± 0.18	4.57 ± 0.19	5.03 ± 1.32
	d214	4.40 ± 0.14	4.35 ± 0.14	4.43 ± 0.03	4.29 ± 0.13
Na (mmol·L^−1^)	d92	145.4 ± 1.2	147.0 ± 1.9	145.0 ± 1.6	143.1 ± 3.5
	d184	144.6 ± 2.1	144.3 ± 1.4	144.2 ± 1.8	142.7 ± 2.4
	d214	145.3 ± 1.2	144.0 ± 2.5	144.8 ± 3.0	144.1 ± 3.6
Cl (mmol·L^−1^)	d92	105.2 ± 1.8	103.4 ± 2.1	103.4 ± 2.0	100.1 ± 3.5 *
	d184	103.5 ± 1.1	102.2 ± 0.8 *	102.3 ± 1.8 *	99.8 ± 1.6 *
	d214	103.9 ± 0.6	104.8 ± 0.4	102.4 ± 0.9	104.9 ± 2.6

Notes: * A significant difference at *p* < 0.05 level compared with the control group. C = control group (distilled water), L = low-dose group (STE 3.75 mg/kg), M = mid-dose group (STE 7.50 mg/kg), H = high-dose group (STE 15.00 mg/kg).

**Table 6:** Summary of serum biochemistry in female rats ($\overset{–}{x}$ ± SD, d92 *n* = 5, d184 *n* = 10, d214 *n* = 5).

Items	Days	C	L	M	H
ALT (nmol·s^−1^·L^−1^)	d92	614 ± 49	656 ± 87	796 ± 250	1093 ± 357 *
	d184	631 ± 71	619 ± 115	879 ± 254 *	1304 ± 441 *
	d214	688 ± 163	740 ± 136	583 ± 121	621 ± 32
AST (nmol·s^−1^·L^−1^)	d92	2466 ± 375	2099 ± 300	2520 ± 392	2422 ± 345
	d184	2529 ± 268	2287 ± 276	2276 ± 486	2478 ± 319
	d214	2329 ± 466	2168 ± 424	2150 ± 307	2262 ± 327
TP (g·L^−1^)	d92	63.2 ± 4.8	59.8 ± 3.5	59.3 ± 3.4	58.2 ± 3.2
	d184	59.7 ± 3.0	58.4 ± 3.0	59.0 ± 2.6	56.8 ± 3.2
	d214	60.8 ± 4.3	60.7 ± 4.4	60.4 ± 4.8	58.4 ± 1.6
ALB (g·L^−1^)	d92	37.7 ± 2.3	35.7 ± 1.5	35.7 ± 1.5	35.4 ± 2.1
	d184	37.6 ± 1.8	36.9 ± 2.0	37.4 ± 2.0	37.0 ± 1.6
	d214	39.3 ± 2.4	37.6 ± 3.1	38.3 ± 3.3	36.6 ± 1.0
TBIL (μmol·L^−1^)	d92	1.49 ± 0.76	1.89 ± 0.83	1.91 ± 0.64	3.27 ± 2.12
	d184	2.88 ± 1.07	3.35 ± 1.10	2.24 ± 1.43	4.24 ± 2.76
	d214	3.02 ± 0.71	2.91 ± 0.57	2.81 ± 0.97	2.22 ± 0.74
GLU (mmol·L^−1^)	d92	6.66 ± 0.39	6.41 ± 0.10	6.69 ± 0.57	6.85 ± 0.84
	d184	7.22 ± 0.42	7.14 ± 0.51	6.99 ± 0.87	6.96 ± 0.52
	d214	7.68 ± 0.64	7.11 ± 0.66	7.40 ± 0.53	7.57 ± 0.78
BU (mmol·L^−1^)	d92	5.94 ± 1.37	7.99 ± 1.18	6.79 ± 0.55	11.05 ± 3.31
	d184	6.30 ± 0.70	6.05 ± 0.60	7.16 ± 1.19	8.52 ± 2.76
	d214	7.06 ± 0.96	7.32 ± 1.27	7.42 ± 0.90	8.39 ± 1.24
CREA (μmol·L^−1^)	d92	33.2 ± 3.7	39.1 ± 6.3	33.8 ± 2.9	42.7 ± 7.0 *
	d184	36.6 ± 3.6	34.2 ± 3.2	33.1 ± 3.6	32.4 ± 5.2
	d214	37.4 ± 2.7	39.1 ± 6.2	38.5 ± 4.5	41.2 ± 5.5
Ca (mmol·L^−1^)	d92	2.3 ± 0.1	2.3 ± 0.1	2.3 ± 0.1	2.3 ± 0.1
	d184	2.4 ± 0.1	2.4 ± 0.1	2.5 ± 0.1	2.5 ± 0.1
	d214	2.7 ± 0.1	2.7 ± 0.1	2.8 ± 0.1	2.7 ± 0.1
P (mmol·L^−1^)	d92	1.75 ± 0.14	1.70 ± 0.08	1.92 ± 0.15	1.80 ± 0.12
	d184	1.70 ± 0.23	1.66 ± 0.19	1.76 ± 0.17	1.78 ± 0.19
	d214	1.36 ± 0.10	1.55 ± 0.14	1.43 ± 0.23	1.57 ± 0.25
TCH (mmol·L^−1^)	d92	1.49 ± 0.18	1.80 ± 0.51	1.79 ± 0.36	1.90 ± 0.24
	d184	1.07 ± 0.26	1.77 ± 0.41 *	2.10 ± 0.46 *	1.73 ± 0.21 *
	d214	0.90 ± 0.31	1.68 ± 0.42 *	1.40 ± 0.47	1.24 ± 0.35
TG (mmol·L^−1^)	d92	0.31 ± 0.03	0.31 ± 0.14	0.37 ± 0.08	0.34 ± 0.08
	d184	0.42 ± 0.05	0.43 ± 0.07	0.50 ± 0.12	0.44 ± 0.06
	d214	0.46 ± 0.06	0.46 ± 0.09	0.48 ± 0.07	0.52 ± 0.22
CK (μmol·s^−1^·L^−1^)	d92	9.43 ± 1.70	6.34 ± 2.10	8.37 ± 2.68	7.16 ± 1.61
	d184	10.00 ± 2.51	8.14 ± 1.76	8.10 ± 3.42	8.83 ± 1.56
	d214	9.89 ± 3.99	8.34 ± 2.17	9.97 ± 3.21	9.47 ± 2.48
LDH (μmol·s^−1^·L^−1^)	d92	29.81 ± 6.15	22.42 ± 6.68	30.52 ± 6.71	27.71 ± 5.51
	d184	27.96 ± 3.17	24.72 ± 4.43	24.15 ± 7.25	25.33 ± 4.21
	d214	25.11 ± 8.58	21.92 ± 4.72	23.70 ± 5.79	24.99 ± 5.70
ALP (μmol·s^−1^·L^−1^)	d92	0.67 ± 0.20	0.66 ± 0.14	0.80 ± 0.34	1.40 ± 0.68
	d184	0.79 ± 0.24	0.69 ± 0.25	0.89 ± 0.42	1.43 ± 0.65
	d214	0.71 ± 0.13	0.77 ± 0.19	0.68 ± 0.12	0.95 ± 0.30
K (mmol·L^−1^)	d92	4.72 ± 0.34	4.59 ± 0.34	4.61 ± 0.28	4.80 ± 0.24
	d184	4.14 ± 0.17	4.18 ± 0.27	4.23 ± 0.18	4.51 ± 0.29 *
	d214	4.15 ± 0.23	4.11 ± 0.21	4.18 ± 0.25	4.17 ± 0.12
Na (mmol·L^−1^)	d92	145.7 ± 0.6	144.4 ± 1.9	145.2 ± 1.3	143.1 ± 4.5
	d184	145.0 ± 1.2	144.3 ± 1.5	144.4 ± 0.9	142.6 ± 3.0 *
	d214	143.5 ± 2.2	144.2 ± 1.9	145.7 ± 2.8	145.0 ± 2.2
Cl (mmol·L^−1^)	d92	107.2 ± 1.4	103.4 ± 0.7	105.2 ± 1.8	101.3 ± 5.2 *
	d184	105.6 ± 1.6	104.8 ± 1.2	103.1 ± 1.5 *	102.3 ± 3.0 *
	d214	107.6 ± 1.4	104.5 ± 0.9 *	104.8 ± 2.3	105.4 ± 0.8

Notes: * A significant difference at *p* < 0.05 level compared with the control group. C = control group (distilled water), L = low-dose group (STE 3.75 mg/kg), M = mid-dose group (STE 7.50 mg/kg), H = high-dose group (STE 15.00 mg/kg).

**Table 7:** Summary of absolute organ weights for male rats ($\overset{–}{x}$ ± SD, d92 *n* = 5, d184 *n* = 10, d214 *n* = 5).

Organ	Days	C	L	M	H
Heart	d92	1.42 ± 0.18	1.36 ± 0.08	1.31 ± 0.18	1.22 ± 0.16 *
	d184	1.58 ± 0.22	1.47 ± 0.20	1.44 ± 0.14	1.33 ± 0.12 *
	d214	1.71 ± 0.22	1.54 ± 0.10	1.57 ± 0.17	1.45 ± 0.12
Liver	d92	10.82 ± 1.28	9.74 ± 0.91	9.40 ± 0.70	6.78 ± 1.09 *
	d184	11.73 ± 2.17	10.78 ± 1.21	10.36 ± 1.30	7.16 ± 1.20 *
	d214	12.75 ± 1.71	11.02 ± 0.94	12.30 ± 3.35	10.14 ± 0.58
Spleen	d92	0.80 ± 0.10	0.68 ± 0.09	0.66 ± 0.08	0.70 ± 0.11
	d184	0.79 ± 0.07	0.76 ± 0.12	0.69 ± 0.11 *	0.62 ± 0.06 *
	d214	0.83 ± 0.16	0.76 ± 0.04	0.85 ± 0.25	0.74 ± 0.10
Lung	d92	1.35 ± 0.08	1.33 ± 0.17	1.30 ± 0.12	1.28 ± 0.20
	d184	1.40 ± 0.15	1.43 ± 0.10	1.38 ± 0.13	1.38 ± 0.14
	d214	1.57 ± 0.13	1.42 ± 0.13	1.45 ± 0.19	1.48 ± 0.10
Kidney	d92	2.69 ± 0.24	2.59 ± 0.27	2.47 ± 0.18	2.24 ± 0.38 *
	d184	2.90 ± 0.53	2.81 ± 0.68	2.75 ± 0.32	2.44 ± 0.21 *
	d214	3.36 ± 0.07	2.99 ± 0.54	2.84 ± 0.29	2.67 ± 0.09
Brain	d92	2.09 ± 0.14	2.04 ± 0.08	2.09 ± 0.09	1.96 ± 0.07
	d184	2.19 ± 0.16	2.16 ± 0.09	2.19 ± 0.07	2.05 ± 0.06 *
	d214	2.16 ± 0.04	2.18 ± 0.06	2.22 ± 0.20	2.08 ± 0.11
Adrenal gland	d92	0.07 ± 0.01	0.06 ± 0.01	0.06 ± 0.01	0.06 ± 0.01
	d184	0.05 ± 0.01	0.06 ± 0.01	0.06 ± 0.01	0.05 ± 0.01
	d214	0.05 ± 0.01	0.05 ± 0.01	0.04 ± 0.01	0.06 ± 0.01
Thymus	d92	0.35 ± 0.10	0.35 ± 0.06	0.26 ± 0.05	0.24 ± 0.02
	d184	0.29 ± 0.14	0.25 ± 0.07	0.22 ± 0.09	0.18 ± 0.04 *
	d214	0.39 ± 0.09	0.22 ± 0.09	0.36 ± 0.07	0.38 ± 0.25
Testis	d92	2.96 ± 0.21	3.01 ± 0.19	3.04 ± 0.15	2.91 ± 0.30
	d184	2.71 ± 0.83	3.07 ± 0.24	3.06 ± 0.24	2.96 ± 0.24
	d214	3.12 ± 0.16	2.96 ± 0.22	3.02 ± 0.19	3.04 ± 0.27
Epididymis	d92	1.37 ± 0.23	1.35 ± 0.16	1.24 ± 0.09	1.31 ± 0.24
	d184	1.21 ± 0.34	1.22 ± 0.15	1.23 ± 0.15	1.22 ± 0.11
	d214	1.30 ± 0.10	1.19 ± 0.17	1.45 ± 0.19	1.24 ± 0.05

Notes: * A significant difference at *p* < 0.05 level compared with the control group. C = control group (distilled water), L = low-dose group (STE 3.75 mg/kg), M = mid-dose group (STE 7.50 mg/kg), H = high-dose group (STE 15.00 mg/kg).

**Table 8:** Summary of absolute organ weights for female rats ($\overset{–}{x}$ ± SD, d92 *n* = 5, d184 *n* = 10, d214 *n* = 5).

Organ	Days	C	L	M	H
Heart	d92	1.00 ± 0.12	1.00 ± 0.10	0.87 ± 0.10	0.67 ± 0.12 *
	d184	0.99 ± 0.11	1.03 ± 0.10	0.89 ± 0.04	0.71 ± 0.10 *
	d214	1.00 ± 0.08	1.01 ± 0.07	1.01 ± 0.11	1.03 ± 0.08
Liver	d92	6.17 ± 0.23	6.28 ± 0.55	6.93 ± 0.44 *	7.49 ± 0.62 *
	d184	6.41 ± 0.58	6.76 ± 0.66	7.71 ± 1.30 *	8.16 ± 0.91 *
	d214	6.34 ± 0.17	6.58 ± 0.32	6.91 ± 0.52	6.70 ± 0.61
Spleen	d92	0.52 ± 0.07	0.52 ± 0.09	0.50 ± 0.03	0.47 ± 0.04
	d184	0.54 ± 0.06	0.57 ± 0.10	0.55 ± 0.11	0.49 ± 0.04
	d214	0.59 ± 0.14	0.52 ± 0.09	0.51 ± 0.04	0.51 ± 0.09
Lung	d92	1.19 ± 0.30	1.11 ± 0.11	1.05 ± 0.11	1.10 ± 0.25
	d184	1.14 ± 0.11	1.14 ± 0.08	1.20 ± 0.09	1.11 ± 0.07
	d214	1.11 ± 0.05	1.17 ± 0.14	1.16 ± 0.10	1.13 ± 0.13
Kidney	d92	1.83 ± 0.15	1.67 ± 0.14	1.73 ± 0.18	1.60 ± 0.21 *
	d184	1.92 ± 0.20	1.91 ± 0.16	1.88 ± 0.16	1.78 ± 0.12 *
	d214	1.86 ± 0.13	1.86 ± 0.08	1.87 ± 0.04	1.88 ± 0.09
Brain	d92	1.91 ± 0.18	1.88 ± 0.08	1.93 ± 0.06	1.78 ± 0.36
	d184	2.02 ± 0.11	2.05 ± 0.06	2.02 ± 0.08	1.96 ± 0.08
	d214	2.05 ± 0.08	2.02 ± 0.09	2.06 ± 0.06	2.03 ± 0.08
Adrenal gland	d92	0.08 ± 0.01	0.08 ± 0.01	0.08 ± 0.01	0.07 ± 0.02
	d184	0.07 ± 0.01	0.07 ± 0.01	0.08 ± 0.01	0.07 ± 0.01
	d214	0.07 ± 0.01	0.07 ± 0.01	0.07 ± 0.01	0.08 ± 0.02
Thymus	d92	0.32 ± 0.02	0.29 ± 0.03	0.25 ± 0.06	0.29 ± 0.06
	d184	0.21 ± 0.06	0.23 ± 0.05	0.24 ± 0.06	0.18 ± 0.05
	d214	0.24 ± 0.02	0.21 ± 0.04	0.21 ± 0.04	0.23 ± 0.04
Testis	d92	0.64 ± 0.18	0.56 ± 0.24	0.53 ± 0.12	0.57 ± 0.16
	d184	0.76 ± 0.20	0.69 ± 0.18	0.68 ± 0.15	0.62 ± 0.23
	d214	0.67 ± 0.26	0.59 ± 0.11	0.74 ± 0.21	0.69 ± 0.12
Epididymis	d92	0.16 ± 0.07	0.12 ± 0.04	0.14 ± 0.04	0.11 ± 0.03
	d184	0.09 ± 0.01	0.11 ± 0.03	0.11 ± 0.02	0.11 ± 0.03
	d214	0.12 ± 0.04	0.10 ± 0.03	0.11 ± 0.04	0.12 ± 0.02

Notes: * A significant difference at *p* < 0.05 level compared with the control group. C = control group (distilled water), L = low-dose group (STE 3.75 mg/kg), M = mid-dose group (STE 7.50 mg/kg), H = high-dose group (STE 15.00 mg/kg).

**Table 9:** Summary of histopathology analyses in rats.

Abnormal Pathology	Day 92 (*n* = 10)				Day 184 (*n* = 20)				Day 214 (*n* = 10)			
	C	L	M	H	C	L	M	H	C	L	M	H
Esophagus keratinized stratified squamous epithelium	1	5	5	6	2	5	8	10	1	2	4	5
Stomach swelling or degeneration or atrophy	0	0	2	3	0	0	0	12	0	0	0	2
Liver inflammatory cell infiltrates or degeneration or necrosis	0	7	6	10	0	10	11	15	0	3	4	2
Lung foam cell foci	0	1	3	4	0	4	6	9	0	1	1	3
Kidney degeneration or swelling or atrophy or inflammatory cells infiltration	0	2	1	7	0	6	5	9	0	0	0	1
Spinal cord degeneration or swelling	0	3	3	2	0	5	3	7	0	0	6	2

Notes: The number indicates the quantity of the cases appeared the pathological changes. C = control group (distilled water), L = low-dose group (STE 3.75 mg/kg), M = mid-dose group (STE 7.50 mg/kg), H = high-dose group (STE 15.00 mg/kg).
